# Fillings vs. "Plugs," et Introducers vs. "Pluggers"

**Published:** 1867-05

**Authors:** Andrew B. Brookins


### ARTICLE VI.

### *Fillings vs. "Plugs," et Introducers vs. "Pluggers.*

By Andrew B. Brookins, D.D.S.

It is a matter of surprise that so many of our first-class operators still continue the use of these inexpressive, inelegant, unprofessional terms.

That "gutter-dentists," as Dr. Atkinson facetiously calls them, who have mechanically attached themselves to our profession, with parasitic tenacity, as the sponge to the sea-girt rock of the Grecian Archipelago, or barnacles to an East Indiaman, should bring along with them barbarism and inelegance, is a matter of no great surprise, being isolated and irresponsible; desiring their patients to be no better informed than themselves, and ignoring as innovations the right use of correct language.

We cannot take up a Dental Catalogue but the words "plugs, pluggers," &c., haunt us from beginning to the conclusion, like the ghost of Hamlet, and one does not feel relieved until he has run his pencil through these intractable "plug-uglies" and writes on the margin the proper representatives.

It is a painful commentary on our profession that proper language cannot be universally used to express proper ideas in the advancement of our professional status.

We have no right to expect the commonalty to be cognizant of professional terms till we educate them, and we cannot do this till we educate ourselves. We cannot give a dollar if we do not possess it.

